# Catching a Deceiver in the Act: Processes Underlying Deception in an Interactive Interview Setting

**DOI:** 10.1007/s10484-016-9339-8

**Published:** 2016-05-18

**Authors:** Sabine Ströfer, Elze G. Ufkes, Matthijs L. Noordzij, Ellen Giebels

**Affiliations:** 1 Department of Psychology of Conflict, Risk and Safety, University of Twente, Enschede, The Netherlands; 2 Department of Cognitive Psychology and Ergonomics, University of Twente, Enschede, The Netherlands

**Keywords:** Deception, Suspect interview, Sympathetic nervous system activity, Electrodermal activity, Stress, Cognitive load

## Abstract

Lying is known to evoke stress and cognitive load. Both form cues to deception and lead to an increase in sympathetic nervous system (SNS) activity. But in reality, deceivers stick to the truth most the time and only lie occasionally. The present study therefore examined in an interactive suspect interview setting, whether deceivers still have clearly diverging cognitive and emotional processes from truth tellers when only having the intention to lie incidentally. We found that deceivers who lied constantly diverge from truth tellers in SNS activity, self-reported cognitive load and stress. Across all interviews, SNS activity correlated stronger with self-reports of cognitive load than stress, which supports the cognitive load approach. Furthermore, deceivers who told the truth and lied on only one crucial question, particularly diverged in self-reported stress from truth-tellers. In terms of SNS activity and self-reported cognitive load, no differences were found. Theoretical and practical implications are discussed.

## Introduction

Previous studies showed that lying evokes more stress and is cognitively more challenging than truth telling (Caso et al. 2005), and that these responses in turn increases physiological arousal (Jung and Lee 2012). Therefore, lying may activate both cognitive as well as emotional cues that can be used for deception detection (DePaulo et al. 2003). For example, studies reveal that people show higher sympathetic nervous system (SNS) activity when lying than when telling the truth (Vincent and Furedy 1992; Zuckerman et al. 1981). However, the crucial point of successfully deceiving others is to control the information one is telling in order to create a false belief (Vrij 2008). The operations to create a false belief therefore do not necessarily have to involve constant and explicit fabrications (Sip et al. 2008). For deception to take place, lying is often not even necessary. Deception may take a variety of forms, including half-truths, vagueness, equivocations, and concealments (Carlson et al. 2004). In fact, in natural situations deceivers stick to the truth as close as possible, and when they do mislead they seldom fabricate information but base deceptive accounts on previous experiences (Leins et al. 2013; Strömwall and Willén 2011).

Truth telling with the intention to lie thus makes up a great part of real-life deceptive attempts. This raises the question whether deceivers still have clearly diverging emotional and cognitive processes from truth tellers when only lying incidentally. The key question therefore may not be whether differences in cognitive and emotional load—and its reflection in the SNS activity (e.g., Fernández et al. 2012)—can be measured depending on whether a specific statement is truthful or not. The question rather should be whether cues to deception already can be measured during the mere intention to deceive—the crucial factor underlying deception (Ambach et al. 2008). The present study addresses this question by comparing interviews based on, respectively, fully deceptive and fully truthful accounts, with accounts wherein people largely tell the truth but have the aim to lie on crucial moments. Specifically, we tested in an interactive interview setting whether, compared to lying and truth telling, the intention to lie already increases self-reported stress levels and cognitive demands as well as physiological responses.

## Processes Underlying Deception

Past work on deception processes demonstrated that lying is often accompanied by both increased stress levels and increased cognitive demand. Cognitive load is often higher during lying than during truth telling, because liars have to engage in more cognitive tasks when lying (Vrij 2008). Examples of such tasks are suppressing the truth while coming up with a plausible alternative statement (Spence et al. 2001; Vrij 2008), inferring what the other is thinking, ‘keeping one’s story straight’ and monitoring and controlling one’s own behavior to avoid creating the impression of lying (Vrij et al. 2006a). Self-report studies indeed show that liars not just feel more nervous, less relaxed and calm than truth tellers but also find the task more strenuous, report being more concentrated, and indicate to monitor their non-verbal behavior more than truth tellers (Hartwig et al. 2007; Strömwall et al. 2006; Watson and Sinha 1993).

The trend in more recent deception literature is to focus on cognitive cues above emotional cues in order to better distinguish deceivers from truth tellers (Vrij et al. 2006a). The advantage of focusing on cognitive cues is that increased levels of cognitive load may exclusively be present during deception, whereas, in most interview settings, stress and tension may be present during lying as well as truth telling (US National Research Council 2003; Vrij 2008). Therefore, emotional indicators may be less reliable cues for detecting deception than cognitive cues (Vrij et al. 2006a). Still, the notion that lying increases cognitive load was until now only supported by interview and free-recall studies in which participants either had to give fully deceptive or fully truthful accounts (Leal and Vrij 2008; Leal et al. 2008; Vrij et al. 2006b, 2012). Considering that in real-life situations, deceivers carefully mix truth and lies, it is important to validate the cognitive load approach in an interview setting, wherein deceivers most of the time stick to the truth and only lie incidentally.

## Processes Potentially Underlying the Intention to Deceive

Arguably, both cognitive and emotional load are not just higher during the act of lying, but already during the mere intention to deceive. That is, several mental processes associated with lying are likely to be active during the entire deceptive attempt, including when telling the truth with the intention to deceive. People for example can be nervous to mislead the other person, or be afraid that their attempt to deceive will be discovered (Vrij 2008), even when no lie has been told yet. But also cognitive processes, not directly related to literally lying, may play a role. For instance, deceivers continuously attempt to control their behavior to appear honest and avoid giving away cues to deception (Buller and Burgoon 1996). Such a motivation can already be present when having the mere intention to deceive but not lying yet. In addition, people with an intention to lie need to monitor the conversation more closely because they constantly have to decide whether they can tell the truth or should lie. This may be especially important when switching from truth telling towards lying, because during lying deceivers are even more aware that the observer pays attention to their behavior (Buller and Burgoon 1996). Because managing these processes involves cognitive effort, we predict that the mere intention to deceive already increases cognitive load.

## Testing the Intention to Deceive in an Interactive Interview Setting

Previous experimental studies showed that, compared to truth telling, the physiological response—measured with electrodermal activity (EDA)—already is higher during truth telling with the intention to lie and that the switch from intention to lie toward lying evoked a peak in SNS activity (Ströfer et al. 2015). However, a disadvantage of physiological processes is that it remains unclear whether increased SNS activity may be due to increased cognitive load, emotional stress, or both (Zuckerman et al. 1981). In the present work we therefore also included self-report measures aimed at making a distinction between how deceivers subjectively experience cognitive load and emotional stress during truth telling with the intention to lie compared to consistent lying or consistent truth-telling.

So far, studies looking at processes underlying deception in interview studies did not combine self-reports with physiological measures (Caso et al. 2005; Vrij et al. 2006b). Moreover, previous interview and free-recall studies surrounding deception did not focus on more realistic deceptions wherein deceivers tell the truth most the time but have the intention to lie on crucial moments. Those studies which did investigate the physiology of deception were of a more experimental nature, having participants responding to (often unrelated) questions, prompted on a computer screen where they either lied or told the truth on specific trials or in blocks of questions (Dionisio et al. 2001; Ganis et al. 2003; Ströfer et al. 2015).

Deception, however, is inherently an interactive process of which person-to-person communication is a fundamental part. In real interrogations for example, police officers determine the number and type of questions, ask follow-up questions and demand elaborations and clarifications (Hartwig et al. 2004). For instance, deception in an interactive setting requires deceivers to be prepared for (unexpected) questions and monitor the other person’s reactions. Together with keeping the dialogue running, this should reveal more natural cues to deception (Miller and Stiff 1993). According to Buller and Burgoon’s (1996) Interpersonal Deception Theory, face-to-face interactions force deceivers to process several tasks simultaneously: For instance, impression management on verbal and non-verbal behavior, and attending their conversation partner to check whether they were believed and managing their emotions while keeping the conversation smoothly. Hence, researchers must come up with designs that mirror interactive processes at play in real-life interviews (Granhag and Hartwig 2008). The current study adds to the literature by answering this call and testing our hypotheses in such an interactive interview setting.

## The Current Study

The aim of the current study was to examine underlying cognitive and emotional load processes during deception when the deceiver has the intention to deceive but only lies incidentally. To do this we created a paradigm, in which deceivers for a great part were required to stick to the truth and we assessed their responses by combining self-reports with a physiological measure. As cover story for the present study served the testing of an ostensible newly developed assessment center test (ACT; Sackett and Dreher 1982). The ACT contained an assignment in which participants had to solve several tasks. In one of the tasks participants were enticed to sign a document which could be seen as fraud. Hereby, a situation was created in which committing a transgression within the ACT was the participant’s own decision and responsibility. We then interviewed participants about this transgression. The study was based on three veracity conditions: In the *intention condition*, participants were advised that the best strategy to approach the interview would be to tell the truth on all questions but to lie about signing the document—a question appearing at the end of the standardized interview. We in addition created a *lie* condition wherein participants lied on all questions, and contrasted the intention and lie conditions with a *truth* condition wherein participants consistently told the truth.

The interview had the same structure as police interviews that build up with the aim to determine whether a suspect is lying or not (Horvath et al. 1994). As such, the interview followed a prescribed script with standard questions. It started broadly, and became continuously more specific regarding the transgression and disclosed the evidence against the participant not until late in the interview (Hartwig 2005; Hartwig et al. 2006). We employed an information gathering interview style, a method based upon rapport and respect, in which interviewers request suspects to give detailed statements about their activities through open questions (Kelly et al. 2013; Vrij et al. 2007).

During the interview we measured the physiological response of the sympathetic nervous system in form of electrodermal activity (EDA). EDA is an indicator for stress and cognitive load (Engström et al. 2005; Heereman and Walla 2011; Hout et al. 2000; Nourbakhsh et al. 2012; Page and Robson 2007; Peter Bankart and Elliott 1974; Shi et al. 2007; Wilson 2002) and forms the most frequently used physiological measure by scholars and practitioners in the field of deception (Vrij 2000). EDA has several advantages over other physiological measures: It directly reflects SNS activity and can be measured unobtrusively within one measurement (Boucsein 2012; Dawson et al. 2007; Wallin 1981).

Moreover, participants’ subjectively experienced cognitive and emotional load during the interview was assessed with self-reports. We took the self-reports directly after the interview, because concurrent assessments during the interview could be obtrusive and influence the behavior under investigation (Kazdin 1979). Also, research has shown that momentary emotion experiences correlate highly with recall-based ratings of emotions (Barrett 1997).

## Hypotheses

In line with earlier studies examining cognitive load during lying (Caso et al. 2005; Vrij et al. 2006b), we expected that the self-reported cognitive load would be higher in the lie compared to the truth condition (Hypothesis 1a). In the lie condition, participants continuously had to make up a false story and come up with deceptive answers. The content of the questions was related, which meant that the deceptive answers had to form a coherent story which should be easier in the truth than in the deception condition, because the truth comes to mind automatically (Walczyk et al. 2003, 2005).

Similarly, we expected self-reported stress to be higher in the lie than in the truth condition (Hypothesis 1b). Participants should be more nervous during lying, both, because they are afraid of not being believed or being excited to mislead the interviewer (a money price was promised to the three best interviewees). Since both, stress and cognitive load lead to an increase in EDA (Engström et al. 2005; Heereman and Walla 2011; Hout et al. 2000; Nourbakhsh et al. 2012; Page and Robson 2007; Peter Bankart and Elliott 1974; Shi et al. 2007; Wilson 2002), we expect EDA to be higher during the lie compared to the truth condition (Hypothesis 1c).

In consideration of that deceivers only lie incidentally in an attempt to deceive, we additionally wanted to test whether the mere intention to lie can be differentiated on self-reported cognitive load, stress and/or physiological responses from truth telling. Theoretically, there are reasons to assume that both, cognitive load and stress are already increased at the foresight of lying. During truth telling with the intention to lie, participants have to decide whether to lie or not, prepare to lie, monitor oneself, the other and their story. They also could experience stress before the actual lie takes place (Ströfer et al. 2015). The more the participants progress through the interview, the more the questions are related to the relevant question ‘Is this your signature?’ (which was the question revealing the evidence). It therefore could be expected that nervousness already increases at these questions, because of the anticipation of the relevant question. Therefore, we expected both self-reported cognitive load and stress to be higher in the intention than in the truth condition (Hypothesis 2a and 2b).

In line with these assumptions and previous findings (Ströfer et al. 2015), we expected that truth telling with the intention to lie evokes higher EDA than ‘honest’ truth telling in the truth condition (Hypothesis 2c). Above that, cognitive load and stress caused by preparing to lie should become most taxing when switching from truth telling to actual lying. We therefore expected that switching from the truth telling toward lying in the intention condition would induce a higher EDA response than switching to the same question in the other two conditions (Hypothesis 3).

## Method

### Participants

We conducted an experiment with 85 graduate students participating in exchange for course credit. Participants randomly were allocated to a veracity condition (Truth, Lie, Intention). Nineteen participants refused to sign the document that served as basis for the experiment and therefore were excluded from the experiment. The data of three participants who did not follow the instructions of the experiment were removed as well, leaving 63 participants for statistical analyses of the self-report data. Participants sometimes inadvertently skipped a self-report question. For this reason, the degrees of freedom reported in the results section sometimes differ between analyses.

Due to technical failures we failed to record EDA data for 7 participants, therefore analyses for physiological responses are based on 56 participants which were equally distributed across conditions (Truth condition: *n* = 19, mean age = 20.37, *SD* = 2.41, range 19–29 years; 12 women; Lie condition: *n* = 18, mean age = 20.88, *SD* = 2.47, range 18–27 years; 13 women; Intention condition: *n* = 19, mean age = 20.89, *SD* = 2.87, range 18–28 years; 13 women). Participants provided written informed consent, and the institutional review board approved the experimental protocol.

### Experimental Design

The experiment consisted of a 3 (veracity condition: truth, lie or intention) × 8 (question type: 1–8) mixed design^1^ Veracity condition was a between subject-factor to which participants were randomly assigned, and question type a within-subject factor. We assessed phasic and tonic electrodermal activity, and self-reported stress and cognitive load as dependent variables.

### Procedure

Participants ostensibly took part as test person for a newly developed assessment center test (ACT; Sackett and Dreher 1982). We informed participants that the purpose of the study was to test a new version of a newly developed assessment center test (ACT) for which we needed volunteers. We also emphasized the advantages of participating: gaining experience in doing an ACT and being in the run for a cash prize awarded to the three best performing participants. We used this cover story to create the opportunity of deception in a more realistic situation. At the start of the experiment we explained participants that the ACT consisted of several exercises and that the three best participants completing these exercises each would win 50 €. In reality, the money was allotted among the participants after the experiment. We further explained that all tasks of the session were relevant for the price and that we would clearly state when the experiment was finished. On average, the experimental sessions lasted for 1.5 hours.^2^ Each session was run by an experiment leader and two confederates: one acting as ‘experiment assistant’ and the other as ‘interviewer’.

Participants were debriefed by e-mail after all study-sessions were conducted. We highlighted that the prize money was used to increase motivation to participate in the study. Also, we explained that EDA measurements only can be interpreted on group level and are not indicative for individual performance, and that we therefore randomly allotted the price money among all participants. Furthermore, participants received feedback on their performance on the in-basket task, which often forms a real part of modern ACT but in our case only served as cover story.

#### In-basket Exercise

The experiment started with an in-basket exercise, which often is part of an assessment center test (Dukerich et al. 1990). Participants were invited to assume the role of a manager of a transport company and to substitute a regular employee who currently was on sick leave. Participants were required to complete four tasks normally executed by the sick employee in 15 min. In the third task participants read a contract that had to be signed by the sick employee—as was indicated by the name of the employee that was already printed on the contract. A note explained that the contract was important for the company and had to be signed urgently. Most of the participants (79 %) signed the contract, since this is an easy and fast solvable problem, and continued with the fourth task. However, signing a document under a wrong name is legally not allowed. This transgression served as input for our deception experiment. Participants sometimes hesitated to sign the document. In this case the investigator attempted to convince the participant to sign the document directly after the ACT task by telling that he himself would sign the document, because this would increase the chance of the money price. Participants (*n* = 19) who after this reminder continued resisting signing the document were excluded from the experiment.

#### EDA Baseline Measurement and Confrontation

After finishing the in-basket exercise, participants were brought to the interview room. In order to get an EDA baseline measure, skin conductance sensors were attached to the participants and we asked them to sit down 5 min and relax and wait for the next task of the experiment. We informed participants that this measure assessed the difficulty of the in-basket test.

After 5 min the experiment leader entered the room again, stating that she reviewed the participant’s output of the in-basket tasks, but that a problem occurred regarding one of the documents. The experiment leader then confronted the participant with the fact that (s)he signed a document (s)he was legally not allowed to sign, and informed that (s)he therefore would be interviewed about this incident.

#### Experimental Manipulation

Directly after confronting participants with their transgression participants received a letter advising on the best approach to behave in the upcoming interview about the transgression. This letter formed the experimental manipulation consisting of three veracity conditions: a truth, a lie and an intention to lie condition. In the *truth* condition, the letter advised participants to tell the truth on all questions, including questions about whether one signed the document. In the *lie* condition, the letter advised to lie on all questions, including questions about whether one signed the document. Finally, in the *intention* condition, the letter advised to tell the truth on all questions but to lie on questions whether one signed the document. We also highlighted the question regarding the signature in the truth and lie condition to prevent that the question about the signature would get special meaning in the intention condition only. Hereby we aimed to prevent differences in prospective memory demands between conditions. Finally, we reminded participants that how well they followed the advice would affect their chances for the price money.

#### Interview and Follow-Up Questionnaire

After the participant finished reading the letter the experiment leader left and the interviewer entered the room. The interview was fully standardized, with the interviewer asking a total of 10 questions in a fixed order (see Table 1). Furthermore, the interviewer was trained to behave similarly in each interview and to ask each question using a neutral intonation. Each interview started with a number of general questions and worked its way up to the key question revealing the evidence: ‘Is this your signature on this document?’ (Question 8). Thereafter the interview ended with two closing questions. During the interview, participants’ EDA was recorded. After the interview the experiment leader entered the room again and asked participants to fill in a final questionnaire assessing their self-reported cognitive load and stress.

**Table 1:** Interview questions

Question	Content
1	Can you tell me about your link with the university? How often and why are you here? What exactly are you doing here?
2	Why did you come to University today?
3	Can you describe step by step what you have done after your entry?
4	Did you encounter other people? Who?
5	Can you describe other additional information?
6	Did you participate in an assessment center test?
7	Have you seen this document before?
8	Is this your signature?
9	Do you want to add something?
10	Was everything clear?

The interview consisted of 10 questions. Questions 9 and 10 were not included in the statistical analyses, since these form the closing part of the interview and were contently not relevant for our experimental manipulation

## Measures

### Self-Reports

#### Cognitive Load

We assessed cognitive load with a scale, consisting of 5 items, α = 0.84. Two items, ‘How difficult was the interview?’ and ‘To what extent did you had to concentrate during the interview?’ were based on items used in a study by Cierniak, Scheiter, and Gerjets (2009). The other three items, ‘How much mental effort did the interview require?’, ‘To what degree the interview was mentally demanding?’ and ‘To what extent did you had to think about the answer of the questions?’ were based on items used in a study by Caso et al. (2005). Participants answered these questions on a 5-point scale ranging from 1 (*very little*) to 5 (*very much*). We created a cognitive load score by aggregating the scores on these 5 items. An explorative factor analysis on these five items (method: maximum likelihood, based on Eigenvalues greater than 1) revealed one underlying factor, explaining 62.82 % of the variance.

#### Stress

Stress was measured with four items, α = 0.88, derived from the Perceived Stress Scale (Cohen et al. 1983). We adjusted the items to the interview situation in our study. The items were ‘To which extent did you feel upset during, or directly after the interview?’, ‘To which extent did you feel nervous during, or directly after the interview?’, ‘To which extent did you feel that the stress during, or directly after, the interview increased to such high levels that you could not let go of it?’ and ‘To which extent did you feel tension during, or directly after the interview?’ (e.g., Giebels and Janssen 2005). All items were measured on 7-point Likert scales ranging from 1 (*not at all*) to 7 (*to a great extent*). We created a stress score by aggregating the scores on these 4 items. An explorative factor analysis on these four items (method: maximum likelihood, based on Eigenvalues greater than 1) revealed one underlying factor, explaining 73.49 % of the variance.

### Skin Conductance

#### Recording EDA

EDA was recorded at 256 Hz and down-sampled to 16 Hz. EDA was measured exodermal (constant voltage) via skin conductance using skin conductance sensors (Thought Technology Ltd., Montreal West, Quebec, Canada), attached to the distal phalanx of the right index and ring fingers (Boucsein 2012). The signal was amplified using ProCompInifiniti amplifier (Thought Technology Ltd.) and was recorded in μS. We informed participants that they were not allowed to take any substances which might affect EDA (such as coffee) either shortly before or during the EDA measurement.

#### Range Correction

Preliminary analyses of the data showed high between-subject variation on tonic EDA recordings due to differences in interview length. To adjust for the inter-individual variance in EDA, we applied a range correction by correcting every recorded data point into proportion (between 0 and 1) to the intra-individual range, using a person’s recorded EDA maximum and minimum (Lykken et al. 1966). To assess participants’ maximum and minimum values we used the recorded data from the baseline through the accusation till the interview end. Since phasic EDA is not time dependent, analyses for phasic EDA were based on the raw data. Our reported descriptive statistics were based on raw data for both, tonic as well as phasic EDA (in μS).

#### Tonic and Phasic EDA

EDA measured over a period of time consists of a slowly drifting tonic signal, which is overlaid by short fluctuations, called skin conductance responses (SCRs or phasic EDA; seen as sharp peaks; Figner and Murphy 2010). Both tonic and phasic measures are interesting with regard to the present study. Tonic EDA can indicate which of our experimental conditions evoked highest arousal generally summarized over all individual questions. Phasic EDA on the other side is more sensitive to abrupt, local, short-living changes. This makes it suitable to indicate how arousal changed through the interview between questions (See Ströfer et al. 2015 for a similar argument).

#### Continuous Decomposition Analysis (CDA)

In order to extract the phasic and tonic data from the raw EDA data, we executed a Continuous Decomposition Analysis using Ledalab (Benedek and Kaernbach 2010) which is an algorithm written in MATLAB. We iterated the parameter optimization three times, which is above the minimal iteration of two recommended by Benedek and Kaernbach (2010). This multi-step deconvolution approach is based upon a physiological model of the SCR shape. The algorithm has several outputs but most importantly reports the continuous phasic and tonic component of the signal.

#### EDA Time Segments

After the CDA analysis we separated the signal into time segments, based on the interview questions. Each segment respectively contained the EDA of a question and the corresponding answer. There is variation in the lengths of each segment, which also differed between persons. To compare EDA between segments the average phasic and tonic EDA activity was calculated for each segment. From this we subtracted the average phasic and tonic EDA activity we respectively measured at the baseline.

## Results

### Self-Reported Cognitive Load and Stress

**Fig. 1:**
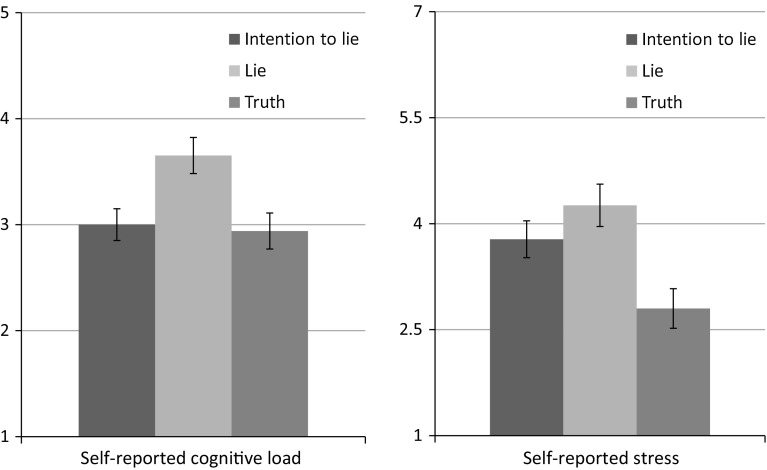
Mean self-reported cognitive load and stress (+ SE) for the three veracity condition (intention to lie, lie, truth). Cognitive load was measured on a 5-point Likert scale and stress on a 7-point Likert scale

Our first aim was to assess differences in self-reported cognitive load and stress between the conditions (see Fig. 1). To analyze these differences, we conducted two ANOVAs with veracity condition (intention to lie/lie/truth) as a between-subject factor on, respectively, self-reported cognitive load and stress. The results revealed a significant main effect of veracity condition on cognitive load *F*(2, 60) = 5.65, *p* = 0.006, η_p_^2^ = 0.16, as well as stress, *F*(2, 58) = 6.67, *p* = 0.002, η_p_^2^ = 0.19. Subsequent simple effect analyses showed that, in line with our predictions, participants reported the lie condition (*M* = 3.65, SE = 0.17) to be more cognitively demanding than the truth condition (*M* = 2.94, SE = 0.17), *t*(37) = 3.53, *p* = 0.005. In the lie condition, self-reported cognitive load was also significantly higher than in the intention condition (*M* = 3.00, *SE* = 0.15), *t*(41) = 2.62, *p* = 0.012, whereas the intention and truth conditions did not differ significantly, *t*(42) = 0.26, *p* = 0.794. Similarly, we found that self-reported stress was significantly higher in the lie condition (*M* = 4.26, *SE* = 0.30) than in the truth condition (*M* = 2.80, *SE* = 0.28), *t*(36) = 3.62, *p* = 0.001. However, self-reported stress was also significantly higher in the intention than in the truth condition, *t*(41) = 2.50, *p* = 0.017, with no significant difference between the lie and intention condition (*M* = 3.78, *SE* = 0.26), *t*(39) = 1.22, *p* = 0.231. These results revealed that in the lie condition participants reported the highest levels of cognitive load as well as stress. For the intention condition, in turn, the results showed that participants report relative low levels of cognitive load (comparable to cognitive load in the truth condition) but still higher levels of stress (comparable to the lie condition). Thus whereas participants in the intention to lie condition did not experience the interview as particularly cognitively demanding, they did experience elevated emotional stress up to the level of participants lying consistently.

### Physiological Responses Across Interviews for Tonic EDA

Our second aim was to investigate the course of the physiological response in SNS activity of someone lying, telling the truth and telling the truth with the intention to lie. We focused on question one to eight, which comprises the questions where participants had to tell the truth (1–7) and had to lie in the intention condition (8). We conducted two mixed factorial ANOVAs with veracity condition (intention to lie/lie/truth) as between-subject factor and question type (1–8) as within-subject factor on tonic and phasic EDA.

For tonic EDA, we found a significant main effect of veracity condition, *F*(2, 53) = 5.72, *p* = 0.006, η_p_^2^ = 0.18 and question type, *F*(7, 371) = 10.91, *p* < 0.001, η_p_^2^ = 0.17. The interaction effect between veracity and question was not significant, *F*(14, 371) = 0.84, *p* = 0.622, η_p_^2^ = 0.03. Simple effect analyses for the main effect of veracity condition revealed that tonic EDA was higher in the lie (*M* = 3.01, *SE* = 0.68) than in the truth condition (*M* = 1.84, *SE* = 0.67), *t*(35) = 1.74, *p* = 0.059, although this effect was not significant, and significantly higher than in the intention condition (*M* = 1.44, *SE* = 0.67), *t*(35) = 4.80, *p* = 0.001. There was no difference in tonic EDA between the intention and truth condition, *t*(36) = 1.30, *p* = 0.15. The pattern for tonic EDA therefore is similar to the pattern we found for self-reported cognitive load.

Simple effect analyses following the main effect of question type, showed that when moving chronologically through the interview, tonic EDA significantly rises from question 1 to 2 *t*(55) = 2.54, *p* = 0.013, remains constant from question 2 to 4, *t*s(55) < 1.30, *p*s > 0.207, and then rises again through question 8, *t*s(55) > 1.93, *p*s < 0.054.

### Physiological Responses Across Interviews for Phasic EDA

**Fig. 2:**
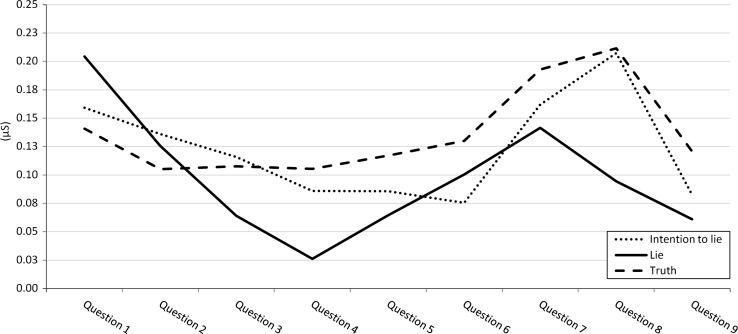
The course of phasic EDA during the interview. Mean phasic EDA from question 1 to 9 (with standard error in parentheses) for the lie condition were 0.20 (0.04), 0.13 (0.04), 0.06 (0.03), 0.03 (0.03), 0.06 (0.04), 0.10 (0.03), 0.14 (0.04), 0.09 (0.04) 0.06 (0.03), for the Intention condition 0.16 (0.04), 0.14 (0.03), 0.12 (0.03), 0.09 (0.03), 0.09 (0.04), 0.08 (0.03), 0.16 (0.04), 0.21 (0.04), 0.08 (0.03) and for the truth condition 0.14 (0.04), 0.11 (0.03), 0.11 (0.03), 0.11 (0.03), 0.12 (0.04), 0.13 (0.03), 0.19 (0.04), 0.21 (0.04), 0.12 (0.03)

In line with the premise that phasic EDA can better discriminate on question level, we found for phasic EDA no significant main effect of veracity condition, *F*(2, 53) = 0.48, *p* = 0.621, η_p_^2^ = 0.018, but a significant main effect of question type, *F*(7, 371) = 8.97, *p* < 0.001, η_p_^2^ = 0.145. Subsequent simple effect analyses revealed that across all three veracity conditions, question 1, 7 and 8 induced the highest peak in EDA. Only on question 8, the relevant question wherein participants were confronted with the evidence, the pattern of phasic EDA between the questions was different within the conditions, which was supported by a significant interaction effect between condition and question type, *F*(14, 371) = 2.02, *p* = 0.015, η_p_^2^ = 0.071. Phasic EDA showed an increase at question 8 in the intention (*M* = 0.21, *SE* = 0.04) and truth condition (*M* = 0.21, *SE* = 0.04) but not in the lie condition (*M* = 0.09, *SE* = 0.04). No significant difference was found between the truth and intention condition, *t*(36) = 0.08, *p* = 0.933. Phasic EDA was higher in both than in the lie condition, *t*s(35) > 2.43, *p*s < 0.035 (see Fig. 2).

### Relationship Between tonic EDA and Self-Reported Stress and Cognitive Load

Our third goal was to gain more insight into the relationship between the self-reported experiences by participants and their physiological responses across the interviews. For this purpose, we took the mean tonic EDA from question one to eight and correlated these with self-reported stress and cognitive load. These analyses revealed a significant correlation between both, tonic EDA and self-reported cognitive load, *r*(54) = 0.50, *p* < 0.001 but also between tonic EDA and self-reported stress, *r*(54) = 0.30, *p* = 0.030. In addition, the correlation between tonic EDA and cognitive load was significantly stronger than that between tonic EDA and stress, *z* = 1.962, *p* = 0.05 (Lee and Preacher 2013; see also Steiger 1980). This suggests that the physiological response of the participants was more strongly related to their subjective experience of how cognitively demanding than of how stressful they experienced the interview.

**Fig. 3:**
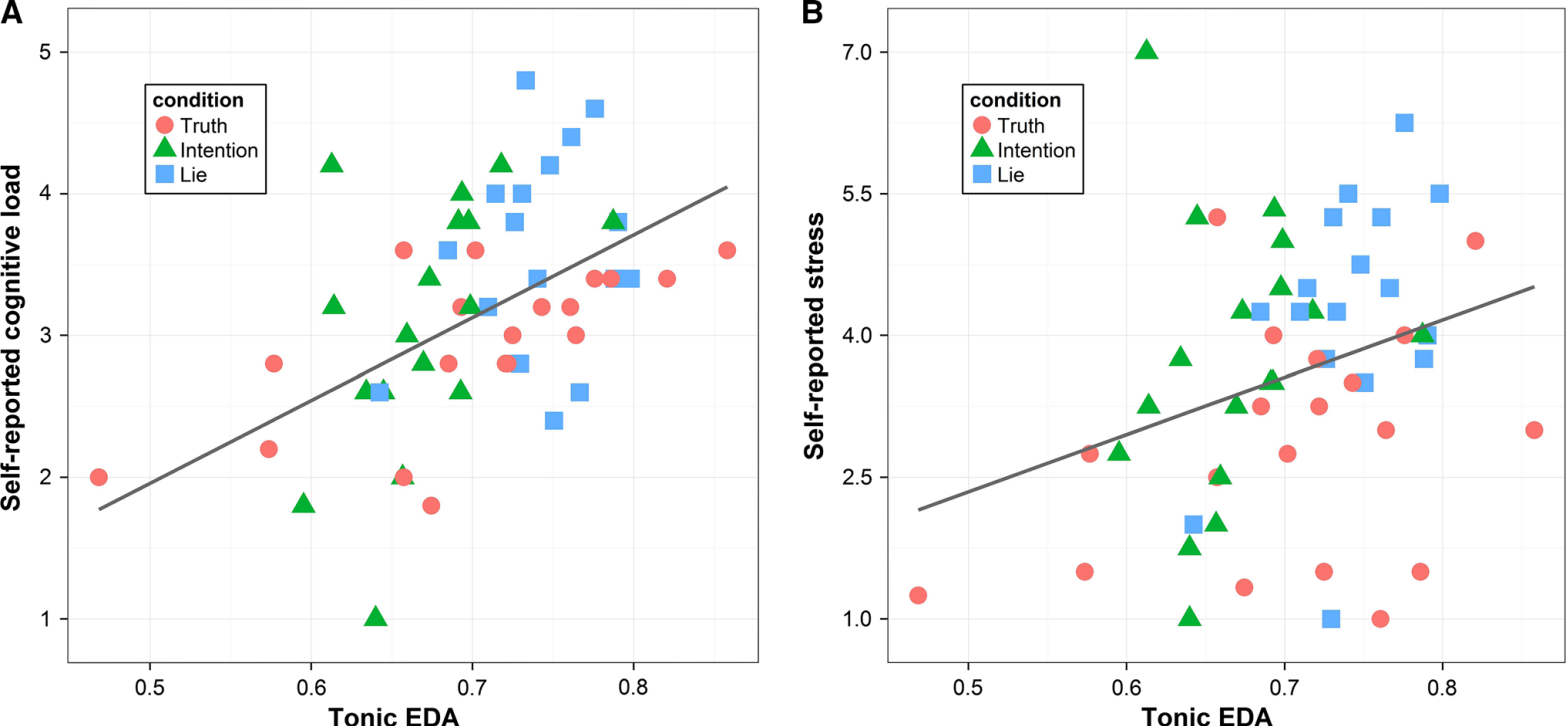
Relationship between tonic EDA (range corrected) and self-reports across all interviews. **a** Relationship between tonic EDA and self-reported cognitive load (measured on a 5-point Likert scale) and **b** relationship between tonic EDA and self-reported stress (measured on a 7-point Likert scale)

When looking at the positive relationship between EDA and self-reported cognitive load in more detail (see Fig. 3, panel a) we see that participants of the lie condition formed a cluster, all scoring high on tonic EDA as well as on self-reported cognitive load. Such a cluster was not present in the other two conditions or for the relation between EDA and stress (see Fig. 3, panel b), where participants showed more variation on both their physiological as well as self-reported responses. This suggests that lying affected all participants in a unique way: it increases both the physiological as well subjective experience of cognitive load during the interview. Truth telling and intention to lie, however, did not have this effect, resulting in more inter-individual variation on how they responded to these measures.

## Discussion

The current study investigated cognitive and affective processes during deception in an interactive interview setting. We specifically focused on the question whether deceivers still have clearly diverging cognitive and emotional processes from truth tellers when only having the intention to lie incidentally. In line with the extant literature, the results showed that participants who consistently lied on all questions had (although not significant) higher physiological responses (e.g., Ströfer et al., 2015), and also reported to experience the interview as more stressful and cognitively demanding, than truth-tellers (e.g., Caso et al., 2005; Vrij et al. 2006a, b). Participants who were required to lie incidentally however, only differed on self-reported stress with truth tellers. Below we will discuss these findings in the light of deception literature, controlled vs. more realistic interview settings, and processes relevant for the intention to deceive.

Previous interview studies all focused on the strict comparison of fully deceptive with fully truthful accounts (Caso et al., 2005; Vrij et al. 1996, 2006b, 2008, 2011, 2012; Warmelink et al. 2013). When deceiving in real life though, people most of the time stick to the truth and only lie incidentally (Leins et al., 2013; Strömwall and Willén 2011). The question therefore arises whether more realistic deceptive attempts, consisting mostly of truth telling and only a few literal lies, still diverge from fully truthful accounts in terms of cognitive load and/or stress. In line with previous research, the current results show that constant lying compared to constant truth telling increases physiological reactions in EDA, which is known to increase with cognitive load and stress (Engström et al., 2005; Hout et al., 2000; Nourbakhsh et al., 2012; Page and Robson 2007; Peter Bankart and Elliott 1974; Shi et al., 2007; Wilson, 2002)—as well as self-reported cognitive load and stress. We assume that the higher self-reports of cognitive load in the lie compared to the truth condition were related to activities directly associated with literally lying, such as suppressing the truth while making up a believable story (Spence et al., 2001; Vrij, 2008), whereas the higher self-reported stress assumable was related to fear and nervousness of not being believed and/or excitement to deceive the other (Ekman, 1985; Vrij, 2008). It is generally assumed that the more deceivers experience stress or cognitive load, the more likely cues to deception (such as increased EDA) would occur (Zuckerman et al., 1981), and the current results further support this notion.

Our main interest however was the intention condition, which resembled real-life deception—wherein truths and lies are mixed—much better. Interestingly, the intention condition could not be differentiated from the truth condition in terms of EDA. Truth telling with and without the intention to lie evoked an equal physiological response pattern. Also, we observed a phasic EDA peak on the relevant question in both conditions (where participants had to lie in the intention and tell the truth in the truth condition).

In first instance, these findings are surprising, because in line with previous work we expected that the mere intention to lie could already evoke higher EDA (Ströfer et al., 2015). The premise was that mental processes associated with deception would be active during the whole deceptive attempt, including the truth telling parts. These could be stress related—for instance, being nervous in the foresight of lying (Vrij, 2008)—as well as having a more cognitive origin—for instance, caused by monitoring oneself, the other, or preparing to lie (Ströfer et al., 2015).

One reason for the similar physiological response patterns when comparing the intention to deceive and truth-telling may be related to our study design. Differences in truth telling with and without the intention to deceive may be too subtle to be detected in interactive scenarios like ours with interpersonal variability. In our study, no interview was exactly the same as the other, even within conditions. It seems thus that in real-life, truth telling with and without the intention to deceive can elicit similar responses, at least on a physiological basis. Another reason for the similar physiological response patterns between the intention to lie and truth-telling conditions is not an absence of these processes in the intention condition. We rather assume that particularly stress related processes, present in the intention condition, underlie ‘honest’ truth telling in a more realistic deception setting as well. Unlike the critic often ascribed to transgression studies, in the present study we compared liars with truth tellers who both transgressed (see for a review DePaulo et al., 2003). Truth telling in the truth condition therefore perhaps was not a neutral act, ‘free’ from feelings of stress, which might explain similar EDA levels during truth telling with and without the intention to lie. Also, this might explain why we found an EDA peak in both condition on the relevant question. Physiological responses to interview questions are not just influenced by veracity alone, but also by how disturbing the questions are experienced (Gudjonsson, 1982): Participants in our truth condition therefore may have had as much stress answering the relevant question than those whose transgressions remained hidden by the lie. This would explain why we found a so called automatic defensive response - a peak in physiological arousal caused by stimuli perceived as aversive or threatening (Campbell et al. 1997; Roelofs et al. 2010) - on the relevant question in both conditions. Truth tellers may have found answering the question “Is this your signature?” as disturbing as deceivers in the intention condition (who lied on this question).

This reasoning matches the fact that whereas previous work did reveal a difference between truth telling with and without the intention to lie on EDA (Ströfer et al., 2015), the current study did not find this difference. That is, in the more experimental studies of Ströfer et al. (2015) participants did not commit a transgression, questions were contently unrelated, and neither had personal relevance nor were embedded in an interactive interview.

Since stress is not unique for people being interviewed who deceive, but also common for people who tell the sincere truth, researchers indeed recently argued to focus on cognitive cues to deception instead (US National Research Council, 2003; Vrij, 2008). However, previous research showed that tonic EDA may be sensitive to stress as well as cognitive load (e.g., Engström et al., 2005; Heereman and Walla 2011; Hout et al., 2000; Nourbakhsh et al., 2012; Page and Robson 2007; Peter Bankart and Elliott 1974; Shi et al., 2007; Wilson, 2002). In our specific study context, higher tonic EDA levels seemed to be more strongly related to self-reported cognitive load than emotional stress. That is, when comparing the experimental conditions, we find similar patterns for self-reported cognitive load and tonic EDA, whereas the pattern for self-reported stress differs: cognitive load and EDA were higher only in the lie condition, while stress was higher in both the lie and intention condition. Moreover, when comparing the relation between tonic EDA and self-reported cognitive load and stress, we found that the correlation was significantly stronger than the correlation between tonic EDA and self-reported stress.

Cognitive load in the lie condition could directly be a result of suppressing the truth while making up a counterfactual statement (Spence et al., 2001; Vrij, 2008). Continuously making up a story may further indirectly affect cognitive load by increasing monitoring behavior, implanted by the deceiver to avoid creating the impression of lying (Buller and Burgoon 1996; Vrij, Fisher, et al., 2006). Previously we reasoned that these latter, indirect processes may not be unique for constant lying, but may be active during the intention to deceive as well. However, having in mind that in the present study the intention and truth condition neither differed in terms of cognitive load nor in EDA, we may conclude that whereas consistently lying is cognitively taxing, having the mere intention to deceive may not be.

Moreover, deception is about creating a belief in others which oneself considers to be untrue (Vrij, 2000). How this belief is created should not matter, because the crucial factor underlying deception is the intention to deceive (Ambach et al., 2008) and this intention should be equally strong for people who consistently are lying and people with the mere intention to deceive. This is also reflected in the present study in terms of affective responses, since the lie and intention conditions were experienced as equally stressful, although in terms of content they were different. Thus, in contrast to the findings with respect to cognitive load, in terms of stress accounts with the intention to deceive are more similar to accounts wherein people are consistently lying. Together these results illustrate the complex nature of cues to deception when deceivers have the intention to deceive but do not lie yet. That is, whereas accounts with the intention to deceive on the one hand may be more similar to truth telling accounts—in both accounts people largely tell the truth—on the other hand they may be more similar to deception accounts—in both type of accounts people have the intention to deceive.

When zooming in on differences in EDA between the different questions, we found that the moment of the critical question triggering participants in the intention condition to lie did induce a high phasic EDA peak. In the lie condition in contrast, phasic EDA showed the opposite effect concerning the question with the signature, with decreased phasic EDA on the relevant question (see Fig. 2). This may be explained by a habituation effect: Frequent lying makes lying easier and frequent truth telling (like in reality and resembled in our intention condition) makes lying more difficult (Hu et al. 2012; Verschuere et al. 2011). Also, the preceding questions demanded much more elaborated answers than the relevant question concerning the signature. Therefore, it is imaginable that participants in the lie condition could have been more cognitively depleted during the interview and were relieved at the ‘relatively’ simple Yes/No question: ‘Is this your signature?’, pointing out the end of the interview.

Although the phasic physiological responses of people who consistently lied, and people who only lied on the relevant question were different, both groups did report to have experienced more stress during the interview than people who only told the truth. It may be possible that self-reported stress in the intention condition was caused by the lie moment itself, whereas in the lie condition stress was experienced across the whole interview. Future studies testing theories on deception processes should use paradigms where truths and lies are mixed to investigate these processes in more detail. Such paradigms may not just qualitatively differ in their underlying processes related to stress and cognitive load; they also reflect real-life interviews better than when participants constantly lie.

## Limitations and Recommendations

The aim of the present study was to find out whether deceivers already diverge from truth tellers when merely have the aim to deceive but do not (have to) lie yet. The answer is yes: Deceptive accounts, consisting mainly of truth telling with only one (crucial) lie differed with truthful ones, but only on self-reported stress and not cognitive load or physiological responses. This supports the emotional load approach which aims to distinguish deceivers and truth tellers by signs of stress, resulting from concerns of being detected (Vrij, 2000). However, the practical gains from the present findings remain restricted as long as these stress differences cannot be measured using more unobtrusive measures than self-reports.

Previous studies surrounding the physiology of the intention to deceive were much more structured (Carrión et al. 2010; Dawson, 1980; Furedy and Ben-Shakhar 1991; Furedy et al. 1988; Ströfer et al., 2015). For the present study we deliberately chose to embed deception in an interview setting resembling real-life interviews as closely as possible. Although this approach increases external validity, topical interviews (such as in the current study) bear other complications such as identifying the source of physiological changes (Cunha et al., 2010). We aimed to overcome this challenge by testing the relationship between self-reported stress and cognitive load and tonic EDA, and found relations between those measures. It remains however difficult to identify the source of physiological changes on question level (local phasic EDA changes) since they cannot be related to self-reports concerning the whole interview.

With regard to further validate the cognitive load approach we encourage future studies to increase the reality of the scenarios against the approach is tested. The current study only found support for the cognitive load approach for fully deceptive accounts. Our findings do not imply that in real-life scenarios where truth and lies are mixed, cognitive load could not play a role at all. In real life, deceivers strategically choose themselves when to lie and when not in a deceptive attempt. This factor could be cognitively demanding, because deceivers have to think when it makes sense to lie. To investigate this, we advise to further develop the paradigm and make it even more realistic. Participants could for instance be given a choice when to lie and when to tell the truth to build up a coherent deceptive story (Sip et al., 2008). Building in factors like ‘free will’ when to lie could make a deceptive attempt cognitively more demanding, even if the truth is told most of the time.

In conclusion, previous deception research mainly focused on finding cues to deception by investigating accounts wherein deceivers are lying consistently. Although, these studies revealed important information on which cues to deception may be useful for deception detection in real-life, they have an important limitation: in real deceptive accounts people almost never consistently lie but stick to the truth as much as possible and only lie on relevant moments in the conversation. The current research shows that the psychological processes during such an intention to lie on the one hand may be similar to processes during lying itself: both create an intention to deceive others which may result in elevated stress levels. On the other hand, processes during accounts with the intention to lie were more similar to truth-telling accounts since both resulted in lower cognitive load than fully deceptive accounts. To discovering cues to deception in real-life conversations, research therefore should focus on both emotional and cognitive indicators of deception.

## Footnotes

1 The original design also contained the number of interviewers as an additional between-subject factor. In half of the interviews a second interviewer was present while merely making notes and actively participating in the interview. Analyses with this factor did not render any significant main or interaction effects. We therefore eliminated number of interviewers from further analyses.

2 Not all parts of the experiment eventually were used for the article. In the experiment participants also filled in a personality questionnaire right at the beginning and did a Stroop-task after the interview. Both data was not used in this article.

## References

[CR1] Ambach W, Stark R, Peper M, Vaitl D (2008). Separating deceptive and orienting components in a Concealed Information Test. International Journal of Psychophysiology.

[CR2] Barrett LF (1997). The relationships among momentary emotion experiences, personality descrptions, and retrospective ratings of emotion. Personality and Social Psychology Bulletin.

[CR3] Benedek M, Kaernbach C (2010). A continuous measure of phasic electrodermal activity. Journal of Neuroscience Methods.

[CR4] Boucsein W (2012). Electrodermal activity.

[CR5] Buller DB, Burgoon JK (1996). Interpersonal deception theory. Communication Theory.

[CR6] Campbell BA, Wood G, McBride T, Lang PJ, Simons RF, Balaban MT (1997). Origins of orienting and defensive responses: An evolutionary perspective. Attention and orienting: Sensory and motivational processes.

[CR7] Carlson JR, George JF, Burgoon JK, Adkins M, White CH (2004). Deception in computer-mediated communication. Group Decision and Negotiation.

[CR8] Carrión RE, Keenan JP, Sebanz N (2010). A truth that’s told with bad intent: An ERP study of deception. Cognition.

[CR9] Caso L, Gnisci A, Vrij A, Mann S (2005). Processes underlying deception: An empirical analysis of truth and lies when manipulating the stakes. Journal of Investigative Psychology and Offender Profiling.

[CR10] Cierniak G, Scheiter K, Gerjets P (2009). Explaining the split-attention effect: Is the reduction of extraneous cognitive load accompanied by an increase in germane cognitive load?. Computers in Human Behavior.

[CR11] Cohen S, Kamarck T, Mermelstein R (1983). A global measure of perceived stress. Journal of Health and Social Behavior.

[CR12] Cunha, M. G., Clarke, A. C., Martin, J. Z., Beauregard, J. R., Webb, A. K., Hensley, A. A., et al. (2010). *Detection of deception in structured interviews using sensors and algorithms.* Paper presented at the SPIE Defense, Security, and Sensing, Orlando, FL, United States.

[CR13] Dawson ME (1980). Physiological detection of deception: Measurement of responses to questions and answers during countermeasure maneuvers. Psychophysiology.

[CR14] Dawson ME, Schell AM, Filion DL, Cacioppo J, Tassinary LG, Berntson GG (2007). The Electrodermal System. Handbook of psychophysiology.

[CR15] DePaulo BM, Lindsay JJ, Malone BE, Muhlenbruck L, Charlton K, Cooper H (2003). Cues to deception. Psychological Bulletin.

[CR16] Dionisio DP, Granholm E, Hillix WA, Perrine WF (2001). Differentiation of deception using pupillary responses as an index of cognitive processing. Psychophysiology.

[CR17] Dukerich JM, Milliken FJ, Cowan DA (1990). In-basket exercises as a methodology for studying information processing. Simulation & Gaming.

[CR18] Ekman P (1985). Telling lies: Clues to deceit in the marketplace, politics and marriage.

[CR19] Engström J, Johansson E, Östlund J (2005). Effects of visual and cognitive load in real and simulated motorway driving. Transportation Research Part F: Traffic Psychology and Behaviour.

[CR20] Fernández C, Pascual JC, Soler J, Elices M, Portella MJ, Fernández-Abascal E (2012). Physiological responses induced by emotion-eliciting films. Applied Psychophysiology and Biofeedback.

[CR21] Figner B, Murphy RO, Schulte-Mecklenburg M, Kuehberger A, Ranyard R (2010). Using skin conductance in judgment and decision making research. A handbook of process tracing methods for decision research: A critical review and user’s guide.

[CR22] Furedy JJ, Ben-Shakhar G (1991). The roles of deception, intention to deceive, and motivation to avoid detection in the psychophysiological detection of guilty knowledge. Psychophysiology.

[CR23] Furedy JJ, Davis C, Gurevich M (1988). Differentiation of deception as a psychological process: A psychophysiological approach. Psychophysiology.

[CR24] Ganis G, Kosslyn SM, Stose S, Thompson W, Yurgelun-Todd DA (2003). Neural correlates of different types of deception: An fMRI investigation. Cerebral Cortex.

[CR25] Giebels E, Janssen O (2005). Conflict stress and reduced well-being at work: The buffering effect of third-party help. European Journal of Work and Organizational Psychology.

[CR26] Granhag PA, Hartwig M, Davies G, Holling C, Bull R (2008). Detecting Deception. Forensic psychology.

[CR27] Gudjonsson GH (1982). Electrodermal responsivity to interrogation questions and its relation to self-reported emotional disturbance. Biological Psychology.

[CR28] Hartwig, M. (2005). *Interrog**ating to detect deception and truth: Effects of strategic use of evidence*.

[CR29] Hartwig M, Anders Granhag P, Strömwall LA (2007). Guilty and innocent suspects’ strategies during police interrogations. Psychology, Crime & Law.

[CR30] Hartwig M, Granhag PA, Strömwall LA, Kronkvist O (2006). Strategic use of evidence during police interviews: When training to detect deception works. Law and Human Behavior.

[CR31] Hartwig M, Granhag PA, Strömwall LA, Vrij A (2004). Police officers’ lie detection accuracy: Interrogating freely versus observing video. Police Quarterly.

[CR32] Heereman J, Walla P (2011). Stress, uncertainty and decision confidence. Applied Psychophysiology and Biofeedback.

[CR33] Horvath F, Jayne B, Buckley J (1994). Differentiation of truthful and deceptive criminal suspects in Behavior Analysis Interviews. Journal of Forensic Sciences.

[CR34] Hout M, Jong P, Kindt M (2000). Masked fear words produce increased SCRs: An anomaly for Öhman’s theory of pre-attentive processing in anxiety. Psychophysiology.

[CR35] Hu X, Chen H, Fu G (2012). A repeated lie becomes a truth? The effect of intentional control and training on deception. Frontiers in Psychology.

[CR36] Jung KH, Lee JH (2012). Cognitive and emotional correlates of different types of deception. Social Behavior and Personality: an international journal.

[CR37] Kazdin AE (1979). Unobtrusive measures in behavioral assessment. Journal of Applied Behavior Analysis.

[CR38] Kelly CE, Miller JC, Redlich AD, Kleinman SM (2013). A taxonomy of interrogation methods. Psychology, Public Policy, and Law.

[CR39] Leal S, Vrij A (2008). Blinking during and after lying. Journal of Nonverbal Behavior.

[CR40] Leal S, Vrij A, Fisher RP, van Hooff H (2008). The time of the crime: Cognitively induced tonic arousal suppression when lying in a free recall context. Acta Psychologica.

[CR101] Lee, I. A., & Preacher, K. J. (2013). Calculation for the test of the difference between two dependent correlations with one variable in common [Computer software]. Available from http://quantpsy.org.

[CR41] Leins DA, Fisher RP, Ross SJ (2013). Exploring liars’ strategies for creating deceptive reports. Legal and Criminological Psychology.

[CR42] Lykken D, Rose R, Luther B, Maley M (1966). Correcting psychophysiological measures for individual differences in range. Psychological Bulletin.

[CR43] Miller GR, Stiff JB (1993). Deceptive communication.

[CR102] National Research Council. (2003). *The polygraph and lie detection*. Committee to Review the Scientific Evidence on the Polygraph. Washington, DC: The National Academic Press.

[CR44] Nourbakhsh, N., Wang, Y., Chen, F., & Calvo, R. A. (2012). *Using galvanic skin response for cognitive load measurement in arithmetic and reading tasks.* Paper presented at the Proceedings of the 24th Australian Computer-Human Interaction Conference, Melbourne, VIC, Australia.

[CR45] Page M, Robson RCA (2007). Galvanic skin responses from asking stressful questions. British Journal of Nursing.

[CR46] Peter Bankart C, Elliott R (1974). Heart rate and skin conductance in anticipation of shocks with varying probability of occurrence. Psychophysiology.

[CR47] Roelofs, K., Hagenaars, M. A., & Stins, J. (2010). Facing Freeze Social Threat Induces Bodily Freeze in Humans. *Psychological Science*, 1575–1581. 10.1177/0956797610384746 20876881

[CR48] Sackett PR, Dreher GF (1982). Constructs and assessment center dimensions: Some troubling empirical findings. Journal of Applied Psychology.

[CR49] Shi, Y., Ruiz, N., Taib, R., Choi, E., & Chen, F. (2007). *Galvanic skin response (GSR) as an index of cognitive load.* Paper presented at the CHI’07 extended abstracts on Human factors in computing systems, San Jose, CA.

[CR50] Sip KE, Roepstorff A, McGregor W, Frith CD (2008). Detecting deception: The scope and limits. Trends in Cognitive Sciences.

[CR51] Spence SA, Farrow TF, Herford AE, Wilkinson ID, Zheng Y, Woodruff PW (2001). Behavioural and functional anatomical correlates of deception in humans. NeuroReport.

[CR100] Steiger JH (1980). Tests for comparing elements of a correlation matrix. Psychological Bulletin.

[CR52] Ströfer S, Noordzij ML, Ufkes EG, Giebels E (2015). Deceptive intentions: Can cues to deception be measured before a lie is even stated?. PLoS One.

[CR53] Strömwall LA, Hartwig M, Granhag PA (2006). To act truthfully: Nonverbal behaviour and strategies during a police interrogation. Psychology, Crime & Law.

[CR54] Strömwall LA, Willén RM (2011). Inside criminal minds: Offenders’ strategies when lying. Journal of Investigative Psychology and Offender Profiling.

[CR55] Verschuere B, Spruyt A, Meijer EH, Otgaar H (2011). The ease of lying. Consciousness and Cognition.

[CR56] Vincent A, Furedy JJ (1992). Electrodermal differentiation of deception: Potentially confounding and influencing factors. International Journal of Psychophysiology.

[CR57] Vrij A (2000). Detecting lies and deceit: The psychology of lying and the implications for professional practice.

[CR58] Vrij A (2008). Detecting lies and deceit: Pitfalls and opportunities.

[CR59] Vrij A, Fisher R, Mann S, Leal S (2006). Detecting deception by manipulating cognitive load. Trends in Cognitive Sciences.

[CR60] Vrij A, Granhag PA, Mann S, Leal S (2011). Outsmarting the liars: Toward a cognitive lie detection approach. Current Directions in Psychological Science.

[CR61] Vrij A, Leal S, Mann S, Fisher R (2012). Imposing cognitive load to elicit cues to deceit: Inducing the reverse order technique naturally. Psychology, Crime & Law.

[CR62] Vrij A, Mann S, Fisher RP (2006). Information-gathering vs accusatory interview style: Individual differences in respondents’ experiences. Personality and Individual Differences.

[CR63] Vrij A, Mann SA, Fisher RP, Leal S, Milne R, Bull R (2008). Increasing cognitive load to facilitate lie detection: the benefit of recalling an event in reverse order. Law and Human Behavior.

[CR64] Vrij A, Mann S, Kristen S, Fisher RP (2007). Cues to deception and ability to detect lies as a function of police interview styles. Law and Human Behavior.

[CR65] Vrij A, Semin GR, Bull R (1996). Insight into behavior displayed during deception. Human Communication Research.

[CR66] Walczyk JJ, Roper KS, Seemann E, Humphrey AM (2003). Cognitive mechanisms underlying lying to questions: Response time as a cue to deception. Applied Cognitive Psychology.

[CR67] Walczyk JJ, Schwartz JP, Clifton R, Adams B, Wei M, Zha P (2005). Lying person to person about life events: A cognitive framework for lie detection. Personnel Psychology.

[CR68] Wallin BG (1981). Sympathetic nerve activity underlying electrodermal and cardiovascular reactions in man. Psychophysiology.

[CR69] Warmelink L, Vrij A, Mann S, Leal S, Poletiek FH (2013). The effects of unexpected questions on detecting familiar and unfamiliar lies. Psychiatry, Psychology and Law.

[CR70] Watson DC, Sinha BK (1993). Individual differences, social arousal and the electrodermal detection of deception. Personality and Individual Differences.

[CR71] Wilson GF (2002). An analysis of mental workload in pilots during flight using multiple psychophysiological measures. The International Journal of Aviation Psychology.

[CR72] Zuckerman M, DePaulo BM, Rosenthal R (1981). Verbal and nonverbal communication of deception. Advances in Experimental Social Psychology.

